# Intratumoral delivery of recombinant vaccinia virus encoding for ErbB2/Neu inhibits the growth of salivary gland carcinoma cells

**DOI:** 10.1186/1479-5876-12-122

**Published:** 2014-05-10

**Authors:** Laura Masuelli, Massimo Fantini, Monica Benvenuto, Pamela Sacchetti, Maria Gabriella Giganti, Ilaria Tresoldi, Paolo Lido, Florigio Lista, Federica Cavallo, Patrizia Nanni, Jeffrey Schlom, Andrea Modesti, Roberto Bei

**Affiliations:** 1 Department of Experimental Medicine, University of Rome ”Sapienza”, Rome, Italy; 2 Department of Clinical Sciences and Translational Medicine, University of Rome “Tor Vergata”, Rome, Italy; 3 Internal Medicine Residency Program, University of Rome “Tor Vergata”, Rome, Italy; 4 Centro Studi e Ricerche Sanità e Veterinaria Esercito, Rome, Italy; 5 Department of Molecular Biotechnology and Health Sciences; Molecular Biotechnology Center, University of Torino, Torino, Italy; 6 Department of Experimental, Diagnostic and Specialty Medicine, University of Bologna, Bologna, Italy; 7 Laboratory of Tumor Immunology and Biology, Center for Cancer Research, National Cancer Institute, National Institutes of Health, Bethesda, MD, USA

**Keywords:** Vaccine, Cancer, Salivary glands, ErbB2/Neu, Vaccinia virus, Intratumor

## Abstract

**Background:**

The antitumor activity induced by intratumoral vaccination with poxvirus expressing a tumor antigen was shown to be superior to that induced by subcutaneous vaccination. Salivary gland carcinomas overexpress ErbB2. Trastuzumab, a monoclonal antibody to ErbB2, was proposed for salivary gland tumors treatment. We explored the effectiveness of intratumoral vaccination with the recombinant vaccinia virus ErbB2/Neu (rV-*neu*T) vaccine in hampering the growth of transplanted Neu-overexpressing BALB-*neu*T salivary gland cancer cells (SALTO) in BALB-*neu*T mice.

**Methods:**

BALB-*neu*T male mice were subcutaneously injected with SALTO tumor cells and intratumorally vaccinated twice with different doses of either rV-*neu*T or V-wt (wild-type). Tumors were measured weekly. The presence of anti-ErbB2/Neu antibodies was assayed by ELISA, immunoprecipitation or indirect immunofluorescence. Biological activity of immune sera was investigated by analyzing antibody-dependent cellular cytotoxicity (ADCC), SALTO cells proliferation and apoptosis, ErbB2/Neu receptor down regulation and ERK1/2 phosphorylation. Anti-Neu T cell immunity was investigated by determining the release of IL-2 and IFN-gamma in T cells supernatant. Survival curves were determined using the Kaplan-Meier method and compared using the log-rank test. Differences in tumor volumes, number of apoptotic cells, titer of the serum, percentage of ADCC were evaluated through a two-tailed Student’s t-test.

**Results:**

rV-*neu*T intratumoral vaccination was able to inhibit the growth of SALTO cancer cells in a dose-dependent manner. The anti-Neu serum titer paralleled *in vivo* antitumor activity of rV-*neu*T vaccinated mice. rV-*neu*T immune serum was able to mediate ADCC, inhibition of SALTO cells proliferation, down regulation of the ErbB2/Neu receptor, inhibition of ERK1/2 phosphorylation and induction of apoptosis, thus suggesting potential mechanisms of *in vivo* tumor growth interference. In addition, spleen T cells of rV-*neu*T vaccinated mice released IFN-gamma and IL-2 upon *in vitro* stimulation with several Neu-specific peptides located in the extracellular domain of Neu sequence.

**Conclusions:**

rV-*neu*T intratumoral vaccination could be employed to induce an efficient antitumor response and reject transplanted salivary gland tumors. Our findings may have important implications for the design of cancer vaccine protocols for the treatment of salivary gland tumors and other accessible tumors using intratumoral injection of recombinant vaccinia virus.

## Background

The hallmark of poxviruses utilization in anti-cancer immunotherapy is their ability to express large foreign genes without significant disruption of the viral genome. This feature offers the possibility of expressing complex eukaryotic sequences or multiple genes in permissive mammalian cells, ensuring correct post-translational modifications [1]. To date, different poxviridae genera have been successfully used as tumor associated antigens vectors in experimental models. Engineered attenuated recombinant vaccinia virus has now been widely employed as a cancer vaccine in a large number of clinical trials as well. The results of these trials demonstrated that recombinant vaccinia virus infection upon vaccination was safe and that a specific humoral or T cell response against the foreign inserted tumor-associated antigen could be induced in several cancer patients [2-13]. Vaccination with recombinant vaccinia virus can be achieved by systemic (subcutaneous, intradermal, or intramuscular) or intratumoral injection [2,4-18]. Recently, it was demonstrated that the antitumor activity induced by intratumoral vaccination with an avipox virus expressing carcinoembryonic antigen (CEA) and multiple co-stimulatory molecules was superior to that induced by subcutaneous vaccination in CEA-transgenic mice [19]. Similarly, we reported that the intramammary gland vaccination with the recombinant vaccinia virus *neu* (rV-*neu*T) vaccine was more effective than the subcutaneous vaccination in inhibiting mammary carcinogenesis in BALB-*neu*T mice [20]. In addition, the intramammary delivery was more effective than the subcutaneous vaccination in eliciting anti-Neu antibodies, increasing anti-Neu IgG2a/G3 isotypes and inducing antibodies able to trigger mammary tumor cells apoptosis and antibody-dependent cellular cytotoxicity [20]. A prerequisite to using intratumoral delivery is the easy access for antigen delivery to the tumor site. Salivary gland tumors as well as head and neck tumors including tongue, floor of the mouth, palate and mandibular mucosa and so on, appear suitable for such vaccine delivery. Salivary gland tumors are a group of heterogeneous lesions which express ErbB2, whose current treatment involves surgery and adjuvant radio(chemo)therapy. However, therapy response rates have been generally poor for these tumors [21]. Recently, given that the high histopatological similarity between salivary ductal and breast carcinomas, Trastuzumab, a humanized monoclonal antibody to ErbB2, has been proposed as a potential therapy for salivary gland tumors treatment. However, active immunization targeting ErbB2 might induce tumor growth inhibition more efficiently than passive immunotherapy based on the generation of an extended memory immune response.

In this study we examined the effectiveness of the rV-*neu*T intratumoral vaccination in hampering the growth of transplanted Neu-overexpressing BALB-*neu*T salivary gland cancer cells (H-2^d^) (SALTO) in BALB-*neu*T mice. In addition, we explored whether the efficiency of vaccination was dependent on the dose of the rV-*neu*T vaccine (10^8^ vs 10^7^ vs 10^6^ pfu). Considering previous demonstration that a potent anti-Neu humoral response is required to prevent mammary tumor growth in BALB-*neu*T vaccinated mice, we investigated the anti-Neu humoral response following rV-*neu*T vaccination as well as the *in vitro* biological activity of immune sera from rV-*neu*T vaccinated mice. Finally, we determined whether rV-*neu*T vaccination elicits anti-Neu T cell immunity.

Our research suggests that intratumoral vaccination using recombinant vaccinia virus could be efficiently employed for the treatment of salivary gland tumors and other accessible tumors.

## Methods

### Antibodies, peptides, reagents and cells

Neu-overexpressing salivary gland cancer cells (H-2^d^) (SALTO) were kindly provided by Prof. Federica Cavallo (University of Turin) and maintained in DMEM containing 20% fetal bovine serum (FBS). SALTO cells were established from salivary carcinoma arising in BALB-*neu*T transgenic male mice hemizygous for p53^172R-H^ transgene driven by the whey acidic protein promoter [22,23]. NIH3T3 cells expressing normal rat Neu (LTR-Neu) have been previously characterized and kindly provided by Dr. Eddi Di Marco (Tumor Institute of Genova) [24]. Renal epithelial cell lines BSC-1 and NIH3T3 cells were purchased by ATCC. BSC-1, LTR-Neu and NIH3T3 were maintained in DMEM containing 10% FBS. Monoclonal antibody anti-Neu Ab4 (PC04) was purchased from Oncogene Science (Cambridge, MA, USA). Rabbit polyclonal anti-Neu antibody (C18), anti-ERK1/2 antibody and monoclonal antibody anti pERK1/2 were purchased by Santa Cruz Biotechnology (Dallas, Texas, USA). Rabbit polyclonal antibody recognizing the activated cleaved caspase-3 was purchased from Cell Signaling Technology (Beverly, MA, USA).

Goat anti-mouse IgG Alexa fluor-488-conjugated antibody and goat anti-rabbit IgG Alexa fluor-594-conjugated antibody were purchased from Life Technologies™ Molecular Probes (Oregon, USA). Protein G-sepharose, sulforhodamine B, Concanavalin A, goat anti-rabbit secondary antibody HRP-conjugated and all the chemicals were purchased by Sigma Aldrich (Milan, IT). Synthetic peptides located in the extracellular (Neu 15.3, aa 66–74; Neu 42, aa 169–183; Neu 98, aa 393–407; Neu 141, aa 566–580; Neu 156, aa 626–640), transmembrane (Neu 166, aa 666–680) domains of rat Neu sequence [NCI, PubMed Accession 1202344A] [25] were previously described [26].

### Poxviruses

The recombinant vaccinia virus encoding the *neu* oncogene was designated rV-*neu*T (vT67RR-1-1, original lot from Therion Biologics Corp: #SC012197). It encodes the full-length activated rat *neu* oncogene [NCI, PubMed Accession 1202344A] [25]. The wild-type control vaccinia virus was designated V-wt (original lot from Therion Biologics Corp: #062797-NYCBH). Therion Biologics Corp. (Cambridge, MA, USA) kindly provided the poxviruses [20].

Expression of recombinant NeuT encoded by rV-*neu*T was detected by Western blotting after infection of BSC-1 or NIH3T3 cells with V-wt or rV-*neu*T. Cells were infected with 10 pfu (plaque forming unit)/cell of poxviruses and cultured at 37°C for 18 h. Cell lysates, protein concentrations and immunoblotting were performed as previously described [27]. Polyclonal anti-ErbB2/Neu antibody was used to detect recombinant NeuT.

### Transgenic BALB-*neu*T mouse colony

Transgenic BALB-*neu*T male mice were routinely mated with BALB/c females (H-2^d^; Charles River, Calco, Italy) in the animal facilities of Tor Vergata University. Progenies were confirmed for presence of the transgene by Polymerase Chain Reaction (PCR) [28]. Mice were bred under pathogen-free conditions and handled in compliance with European Union and institutional standards for animal research.

### Recombinant vaccinia *neu* vaccination protocol

The protocol of vaccination was approved by the Ethical Committee of the University of Rome “Tor Vergata” and submitted to the Italian Health Department.

Six to 8 weeks old BALB-*neu*T male mice were subcutaneously injected in the right flank with 0.2 ml suspension containing 1x10^6^ SALTO cells in phosphate-buffered saline (PBS). When mice presented a palpable tumor mass around 300 mm^3^ (mean tumor volume 281–356 mm^3^), were intratumorally vaccinated with either rV-*neu*T or V-wt and boosted two weeks later.

Viruses were diluted in PBS such that the dose was delivered in 100 μl. Mice were immunized twice. BALB-*neu*T received for each vaccination a dose of 10^8^ pfu of either rV-*neu*T (n. 9 mice) or V-wt (n. 9 mice), a dose of 10^7^ pfu of either rV-*neu*T (n. 8 mice) or V-wt (n. 6 mice) and a dose of 10^6^ pfu of either rV-*neu*T (n. 6 mice) or V-wt (n. 6 mice).

### Analysis of antitumor activity *in vivo*

Tumor growth was monitored weekly until tumor-bearing mice were sacrificed when tumor exceeded 20 mm diameter. Tumors were measured by a calliper in two dimensions and the volumes were calculated using the formula: width^2^ × length/2 [29].

### Antibody immunity following vaccination with rV-*neu*T

Sera from vaccinated BALB-*neu*T mice were collected prior to vaccination and 7 days after the final boost. The presence of antibodies reactive to p185 Neu was assayed using NIH3T3, LTR-Neu and SALTO cells by enzyme-linked immunosorbent assay (ELISA) or immunoprecipitation following western blotting as previously described [30-32]. For ELISA, individual rV-*neu*T mouse serum at different dilutions (1:1000, 1:2500, 1:12500) was assayed against LTR-Neu and NIH3T3 control (5×10^4^ cells/well). The specific absorbance of each sample was calculated by subtracting LTR-Neu absorbance from that of NIH3T3 cells. Antibody titer was estimated as the highest immune serum dilution generating a specific absorbance of 0.5 at 492 nm. Sera titer is the mean value of individual serum titers [33]. Individual serum samples from mice receiving rV-*neu*T were randomly chosen. Individual V-wt mouse serum was assayed at 1:250 dilution. Immunoglobulin subclasses were determined by ELISA using a Mouse Typer Isotyping Kit (Bio-Rad, Richmond, CA, USA) using individual serum of rV-*neu*T vaccinated mice as previously described [29,33].

For immunoprecipitation, cells were lysed in RIPA buffer containing 1% Triton X-100, 0.5% deoxicolate, 0.1% SDS, 20 mM Tris pH 7.5, 150 mM sodium chloride, proteases and phosphatases inhibitors. Protein concentration was determined using the Bradford protein assay (Bio-Rad, Richmond, CA, USA). Equal amounts of total proteins were immunoprecipitated using sera derived from different animals immunized with rV-*neu*T or V-wt, and Protein G-sepharose overnight at 4°C. The immunoprecipitates were washed three times with RIPA buffer, boiled at 95°C and centrifuged to remove sepharose beads. The immunoprecipitates were separated by SDS-PAGE and transferred into nitrocellulose membrane [30]. After blocking with 5% non-fat milk, the membrane was incubated with polyclonal anti-neu antibody C18 (Santa Cruz Biotechnology Inc. Dallas, Texas, USA). After washing, the membranes were incubated with goat anti-rabbit secondary antibody HRP-conjugated. The antigen-antibody binding was visualized by chemiluminescence using SuperSignal West Pico Chemiluminescent Substrate kit (Pierce, IL, USA) [34].

### Biologic activity of vaccinated mouse immune sera *in vitro*

Antibody-dependent cellular cytotoxicity (ADCC) was investigated as previously described [26,35]. BALB-*neu*T SALTO tumor cells (5×10^3^ cells/well) were used as targets, while spleen cells from normal BALB/c mice were used as effectors at 50:1. Dilutions (1:10, 1:20, 1:40) of sera pooled from four mice vaccinated with 10^8^ pfu rV-*neu*T or V-wt were assayed. Percentage of specific lysis was calculated as described [36]. The results represent average percentage of cytotoxicity of two independent experiments. Four randomly chosen serum samples were pooled each time and used for two independent experiments.

For cell proliferation of BALB-*neu*T SALTO tumor cells, immunoglobulins (Igs) from 10^8^ rV-*neu*T and V-wt pooled sera were purified by protein G and dialyzed against PBS. Purity was determined by SDS-PAGE and Coomassie blue staining. SALTO cells (2.5x10^4^ cells/well) were incubated in serum-free DMEM containing 0.2% BSA containing Igs (20 or 10 μg/ml). Igs were replenished every 24 h. All treatments were performed in triplicate. Survival of cells was assessed by the Sulforhodamine B cell proliferation assay [37]. The percent change in relative cell number was calculated as described by Yip *et al.*[38].

To analyze the ability of serum antibodies to induce down regulation of ErbB2/Neu receptor on SALTO tumor cells, indirect immunofluorescence under native conditions was performed. Briefly, cells were detached by incubation with 0.02% EDTA in PBS and incubated with purified Igs derived from rV-*neu*T or V-wt vaccinated mice at 4°C for 2 hours. After washes with cold PBS, cells were labelled with goat anti-mouse IgG Alexa fluor-488-conjugated antibody (Life Technologies™ Molecular Probes, Oregon, USA) for 1 hour on ice, washed and immediately observed with an Olympus BX51 microscope; I.A.S. version 007 000 Deconvolution 2D software was used to deconvolve z series images of stained native cells [39]. To analyze the ability of Igs to induce receptor down regulation, SALTO tumors cells were incubated in complete medium for 1 hour at 37°C in a CO_2_ incubator after immunolabeling with Igs from rV-*neu*T or V-wt vaccinated mice and goat anti-mouse-fluorescent antibody. SALTO cells were then observed and analyzed as above.

For western blotting analysis of ERK1/2, 40 μg of V-wt or rV-*neu*T Igs chronically treated (see above) SALTO cell lysates were separated by SDS-PAGE and processed for western blotting as previously described using anti-ERK1/2 and anti-pERK1/2 specific antibodies [37,40]. The intensity of the bands obtained in two independent experiments was quantified using the ImageJ software after blot scanning, and densitometric ratios between the phosphorylated and total levels of each protein were calculated.

For detection of programmed cell death, SALTO cells (2.5×10^4^ cells/well) were incubated in serum-free DMEM containing 0.2% BSA containing Igs (10 μg/ml) from rV-*neu*T or V-wt vaccinated mice for 72 hours. Igs were replenished every 24 h. Cells were fixed in 4% formaldehyde for 15 min and after washing they were incubated with the polyclonal anti-activated caspase-3 antibody for 1 h. After washing, cells were labelled with goat anti-rabbit IgG Alexa fluor-594-conjugated antibody (Life Technologies™ Molecular Probes, Oregon, USA) for 60 min [26]. After washing, cells were incubated with 0.1 μg/ml Hoechst 33342 and mounted under a coverslip in glycerol. Staurosporine at 1 μM incubated for 24 h was used as positive control. The percentage of apoptotic cells was calculated by determining the activated caspase-3 positive cells/total cells evaluating five randomly chosen microscopic fields. Count of apoptotic cells was done in a blinded fashion.

### IL-2 and IFN-γ release assay

Spleen cells from BALB-*neu*T vaccinated mice were harvested 7 days after the final vaccination as previously described [26]. Spleen mononuclear cells (2×10^6^/well in 24-well plates) were incubated with Concanavalin A (ConA, 2 μg/ml), several Neu peptides (10 μg/ml) or a control gag peptide. Neu peptides were selected based on immunoreactivity *in vitro* with lymphocytes from BALB-*neu*T mice vaccinated with recombinant adenovirus, LTR-Neu and rV-*neu*T [20,26,41]. IL-2 and IFN-γ release into the supernatant was measured using an enzymatic immunocapture assay (Quantikine, R&D Systems, Minneapolis, MN, USA).

### Statistical analysis

Mean and standard deviation describes continuous variables. Survival curves were determined using the Kaplan-Meier method and compared by the log-rank test (Mantel-Cox). Differences in tumor volumes, number of apoptotic cells, titer of the serum, percentage of ADCC, intensity of immunoreactive bands were evaluated by a two-tailed Student’s t-test. Statistical associations were considered significant at p-values ≤0.05.

## Results

### Expression of recombinant NeuT encoded by rV-*neu*T

**Figure 1:**
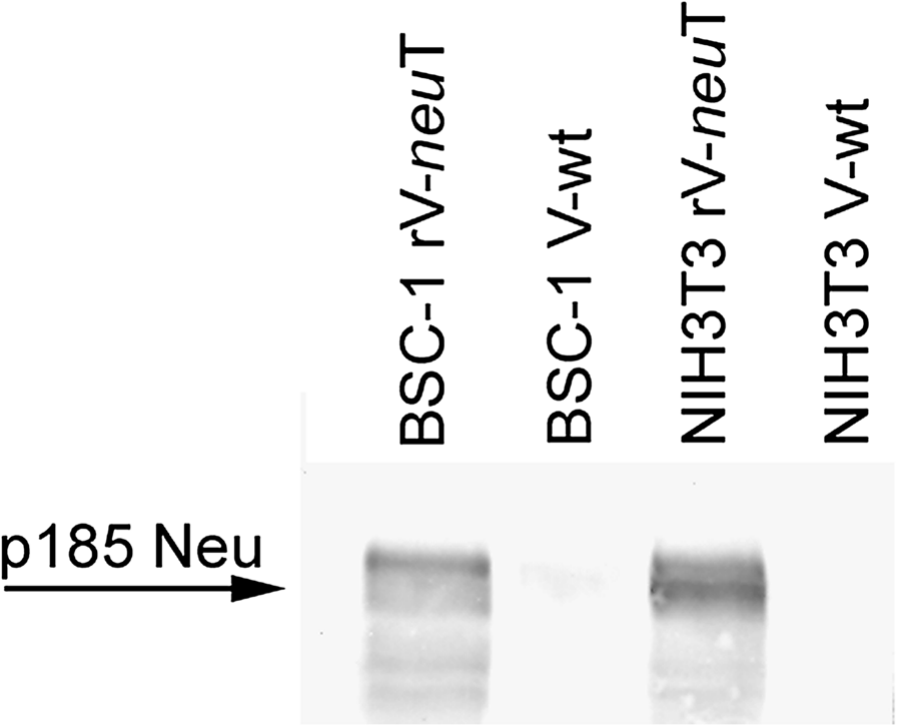
**Expression of recombinant NeuT by rV-*neu*T infected BSC-1 and NIH3T3 cells.** Cells were infected with 10 pfu (plaque forming unit)/cell of poxviruses and cultured at 37°C for 18 hours. Western blotting analysis was performed by using rabbit polyclonal anti-Neu antibody (C18).

Expression of recombinant NeuT encoded by rV-*neu*T was detected by Western blotting after rV-*neu*T infection of BSC-1 and NIH3T3 cells. As shown in Figure 1, polyclonal anti-HER-2/neu antibody detected a 185 kDa protein product on rV-*neu*T-infected BSC-1 and NIH3T3 cells but not in V-wt-infected cells.

### Inhibition of tumor growth by recombinant vaccinia *neu* (rV-*neu*T) vaccine

To determine whether the vaccination with rV-*neu*T was able to induce inhibition of the growth of transplanted p185 Neu positive tumor (SALTO) and whether it was dependent on rV-*neu*T doses, BALB-*neu*T male mice were challenged in the right flank with 1x10^6^ SALTO cells, and immunized twice intratumorally with three different doses of rV-*neu*T or V-wt (10^8^, 10^7^, 10^6^ pfu). Vaccinations were performed at two weeks interval.

**Figure 2:**
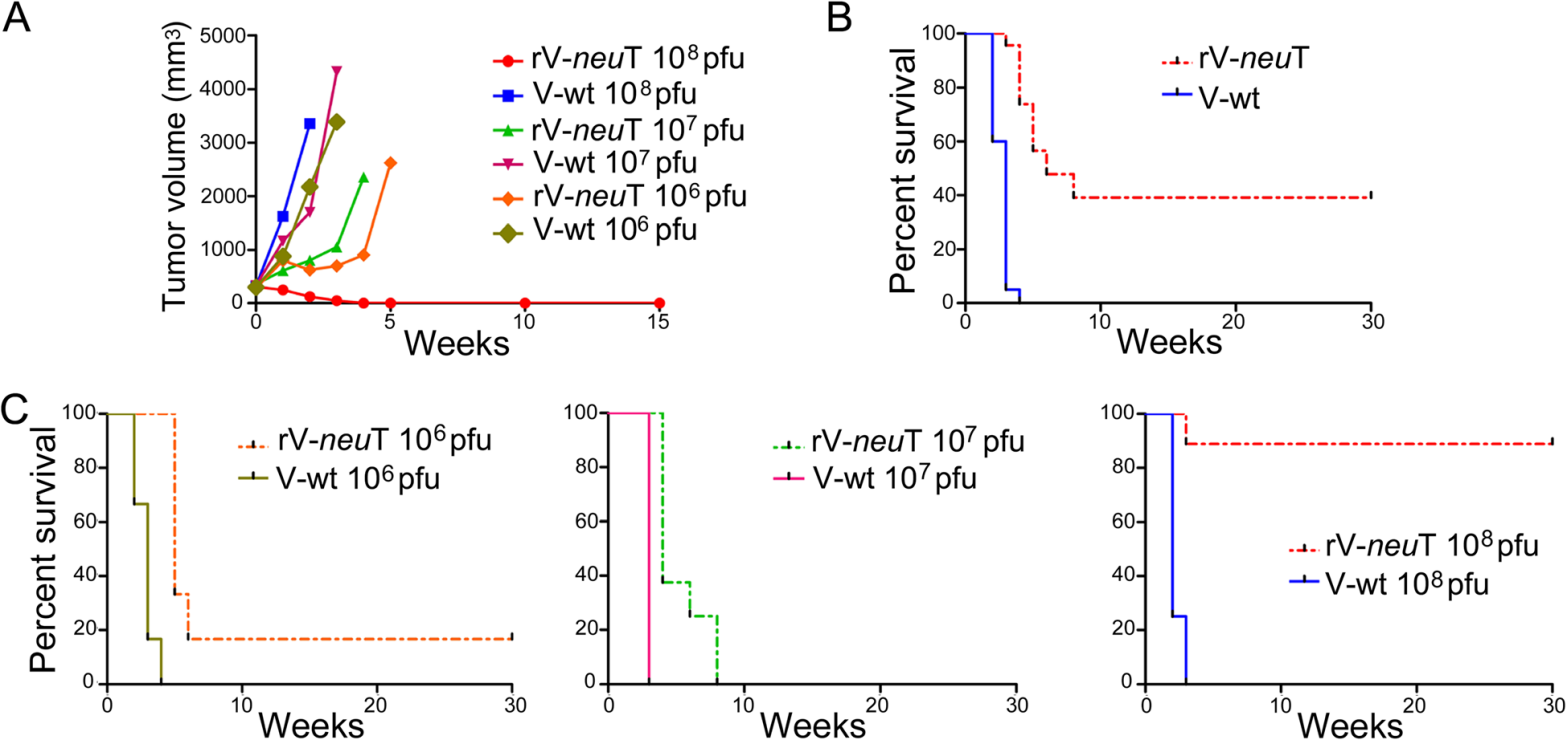
**Inhibition of transplantable salivary gland (SALTO) tumor cells growth *in vivo *by rV-*neu*T vaccination.** Panel **A**: Differences in tumor volume between V-wt and rV-*neu*T vaccinated BALB-*neu*T mice after different doses of vaccination (10^8^ pfu, 10^7^ pfu and 10^6^ pfu). Panel **B**: Differences in mean survival time of rV-*neu*T- and V-wt-vaccinated mice independently of the dose of vaccination. Panel **C**: Differences in mean survival between V-wt and rV-*neu*T vaccinated BALB-*neu*T mice after different doses of vaccination (10^8^ pfu, 10^7^ pfu and 10^6^ pfu). Numbers of vaccinated mice are reported in “Materials and methods”.

Vaccination with rV-*neu*T at the dose of 10^8^ pfu was able to induce regression of transplanted tumors (Figure 2), and to elicit an immunological memory that protected mice against a second injection of SALTO cells (data not shown). When considering the effectiveness of the rV-*neu*T vaccine independently of dose, the mean survival time of mice vaccinated with rV-*neu*T versus those receiving the V-wt was 14.8 versus 2.63 weeks (p < 0.0001) (Figure 2, Panel B).

The 10^8^ pfu dose vaccination was started when mean tumor volume was 307 mm^3^ (9 mice) and 321 mm^3^ (9 mice) in rV-*neu*T or V-wt vaccinated-mice, respectively. Two weeks after the first vaccination mean tumor volume of V-wt vaccinated mice reached 3351 mm^3^ while mean tumor volume of rV-*neu*T vaccinated mice was 123 mm^3^ (p < 0.001) (Figure 2, Panel A). At this stage 1/9 rV-*neu*T vaccinated mice was tumor free. One mouse of this group died for unknown reason although the tumor volume was diminishing after the first vaccination. Four weeks after the first vaccination 5/9 rV-*neu*T vaccinated mice were tumor free and two weeks later 8/9 rV-*neu*T vaccinated mice were tumor free and remained in this status until the 30^th^ week. Conversely, all V-wt vaccinated mice were sacrificed for exceeded tumor volume or spontaneously died at the third week after the first vaccination (p < 0.001) (Figure 2, Panel C). Overall, the mean survival time of mice vaccinated with 10^8^ pfu rV-*neu*T versus those receiving the 10^8^ pfu V-wt dose was 27 versus 2.25 weeks (p < 0.0001) (Figure 2, Panel C).

The 10^7^ pfu dose vaccination was started when mean tumor volume was 356 mm^3^ (8 mice) and 332 mm^3^ (6 mice) for rV-*neu*T and V-wt vaccinated mice, respectively. Three weeks after the first vaccination mean tumors volume was 1052 mm^3^ and 4319 mm^3^ in rV-*neu*T e V-wt vaccinated mice, respectively (p < 0.001) (Figure 2, Panel A). Four mice vaccinated with rV-*neu*T were sacrificed four weeks after the first vaccination for exceeded tumor size and one mouse died, while all V-wt vaccinated mice were sacrificed within the fourth week after the first vaccination (p < 0.001). Only 2/8 mice of the rV-*neu*T vaccinated group were still alive at the 7^th^ week but they were sacrificed within the 8^th^ week (Figure 2, Panel C). The mean survival time of mice vaccinated with 10^7^ pfu rV-*neu*T versus those receiving the 10^7^ pfu V-wt dose was 5.25 versus 3 weeks (p = 0.0003) (Figure 2, Panel C).

Finally, the 10^6^ pfu dose vaccination was started when mean tumor volume was 281 mm^3^ (6 mice) and 298 mm^3^ (6 mice) for rV-*neu*T and V-wt, respectively. Two weeks after the first vaccination mean tumor volume was 624 mm^3^ and 2167 mm^3^ in the group of mice vaccinated with rV-*neu*T and V-wt, respectively (p = 0.0096) (Figure 2, Panel A). Two out of six mice vaccinated with V-wt were sacrificed at this stage. At the third week after vaccination 3/6 V-wt vaccinated mice were sacrificed while 1/6 rV-*neu*T vaccinated mice was tumor free (mean tumor volume: 3385 vs 699 mm^3^ for the vaccination with V-wt and rV-*neu*T, respectively; p = 0.0009). Only 1/6 mice of the group vaccinated with V-wt was alive four weeks after the first vaccination. Conversely 6/6 rV-*neu*T vaccinated mice (mean tumor size: 901 mm^3^) were alive at this stage. At the 7^th^ week, only 1/6 rV-*neu*T vaccinated mice was alive and remained tumor free until the 30^th^ week (Figure 2, Panel C). The mean survival time of mice vaccinated with 10^6^ pfu rV-*neu*T versus those receiving the 10^6^ pfu V-wt dose was 9.33 versus 2.83 weeks (p = 0.0006) (Figure 2, Panel C).

Overall, when comparing the survival of BALB-*neu*T mice upon vaccination it was observed that the risk of growth of SALTO tumor cells in the rV-*neu*T vaccinated group was 0.04 in comparison to V-wt vaccinated group. In addition, the dose of the vaccine significantly affected mice survival (Table 1). The risk of developing tumors in the 10^6^ pfu and 10^7^ pfu rV-*neu*T vaccinated groups was 10.26 and 14.05 in comparison to the 10^8^ pfu rV-*neu*T vaccinated group (Table 1). No difference was found between the 10^7^ and 10^6^ pfu rV-*neu*T vaccination.

**Table 1:** **Comparison of BALB-*neu*** **T mice survival after rV-*neu*** **T and V-wt vaccination by log-rank (Mantel-Cox) test**

**Variable**	**Contrast**	**Hazard ratio**	**95% hazard ratio confidence limits**		***p *** **value**
			**Lower**	**Upper**	
Vaccine	rV-*neu*T vs V-wt	0.04	0.014	0.11	< 0.0001
	rV-*neu*T 10^8^ pfu vs V-wt 10^8^ pfu	0.035	0.007	0.19	< 0.0001
	rV-*neu*T 10^7^ pfu vs V-wt 10^7^ pfu	0.022	0.003	0.18	0.0003
	rV-*neu*T 10^6^ pfu vs V-wt 10^6^ pfu	0.04	0.006	0.25	0.0006
Dose	rV-*neu*T 10^7^ pfu vs rV-*neu*T 10^8^ pfu	14.05	3.05	64.66	0.0007
	rV-*neu*T 10^6^ pfu vs rV-*neu*T 10^8^ pfu	10.26	1.65	63.63	0.012
	rV-*neu*T 10^6^ pfu vs rV-*neu*T 10^7^ pfu				NS

NS: not significant.

These results suggest that rV-*neu*T intratumoral vaccination is able to induce inhibition of the growth of transplanted salivary gland Neu positive tumor cells and that the effect of vaccination is dose-dependent. The lower doses (10^6^ e and 10^7^ pfu) were able to induce in rV-*neu*T vaccinated mice only a delay in SALTO tumor cells growth as compared to V-wt vaccinated mice. In this regard, the mean survival time of mice vaccinated with 10^8^ pfu rV-*neu*T versus those receiving the 10^7^ pfu rV-*neu*T and 10^6^ pfu rV-*neu*T doses was 27 versus 5.25 weeks (p = 0.0007) and 9.33 (p = 0.012) weeks, respectively (Figure 2, Panel C).

### Anti-Neu humoral response following rV-*neu*T vaccination

Previous studies reported that anti-Neu humoral response is required to inhibit mammary tumor growth in BALB-*neu*T vaccinated mice [35]. Antibody response to p185 Neu was quantitatively and qualitatively evaluated by immunoprecipitation following western blotting, ELISA and immunofluorescence in order to determine whether differences in humoral response existed between rV-*neu*T or V-wt administration before and after vaccination.

Specific anti-Neu reactivity in sera from rV-*neu*T vaccinated mice was visualized by immunoprecipitation followed by western blotting by using an anti-Neu specific antibody, and LTR-Neu and SALTO cells as antigen source.

**Figure 3:**
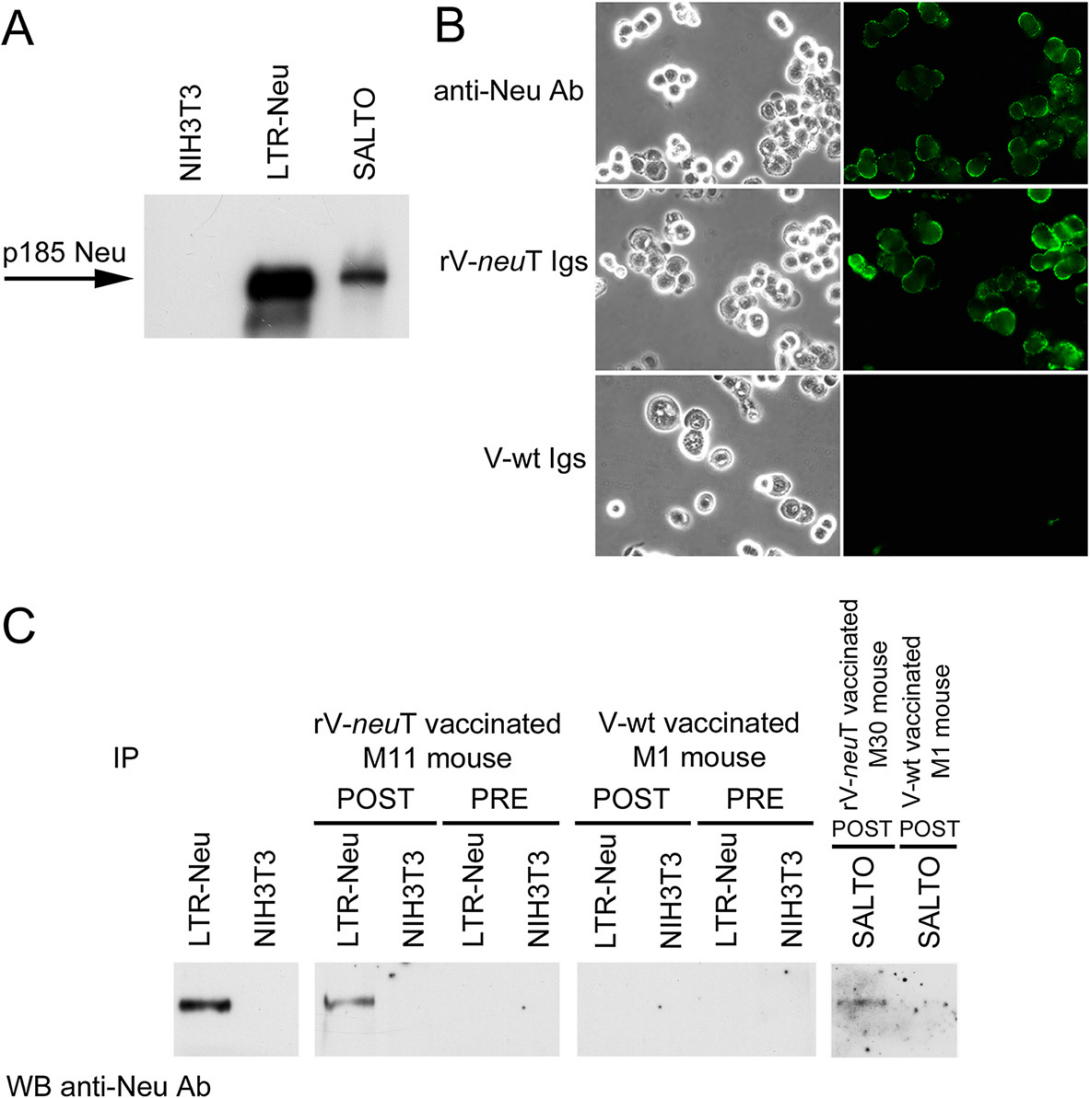
**Anti-Neu humoral response following rV-*neu*T vaccination.** Panel **A**: p185 Neu expression in NIH3T3 cells encoding normal rat Neu (LTR-Neu) and in Neu-overexpressing BALB-*neu*T salivary gland cancer (SALTO) cells by western blotting. A specific polyclonal anti-Neu (C18) antibody was used. NIH3T3 cells were used as negative control. Panel **B**: Igs from rV-*neu*T vaccinated mice recognize p185 Neu expressed on the surface of SALTO tumor cells. Immunofluorescence was performed using purified Igs (1μg/ml) of BALB-*neu*T mice vaccinated with rV-*neu*T or V-wt. The specific monoclonal antibody anti-Neu Ab4 was used as positive control. Olympus BX51 microscope and I.A.S. version 007 000 Deconvolution 2D software were used to deconvolve z series images of stained native cells. Original magnification x400. Panel **C**: Serum antibody response of mice upon vaccination with rV-*neu*T. Numbering identifies immune sera of individual mice. Mouse pre-immune or immune sera were collected prior to vaccination or one week after the second immunization and employed in immunoprecipitation of Neu from LTR-Neu or SALTO tumor cells. p185 Neu specificity was visualized by immunoblotting analysis using receptor-specific polyclonal antibody of immunoprecipitates and compared to direct immunoblotting of LTR-Neu lysate as positive control, or NIH3T3 as negative control.

The expression of p185 Neu in LTR-Neu and in SALTO cells was analyzed by western blotting. As shown in Figure 3, Panel A, NIH3T3 fibroblasts did not express p185 Neu, while LTR-Neu and SALTO cells showed high levels of expression of p185 Neu. Specific antibody response to Neu was qualitatively evaluated by indirect immunofluorescence and immunoprecipitation analysis. Mouse pre-immune or immune sera were collected prior to vaccination or one week after the final boost. SALTO cells were incubated with purified Igs derived from rV-*neu*T or V-wt BALB-*neu*T vaccinated mice followed by labeling with goat anti-mouse IgG Alexa fluor-488-conjugated antibody. Figure 3, Panel B shows representative membrane staining of SALTO cells by Igs form rV-*neu*T vaccinated mice similarly to that of monoclonal anti-Neu antibody Ab4. Conversely, Igs derived from V-wt vaccinated Balb-*neu*T mice or pre immune serum (data not shown) did not bind SALTO cells. Sera were employed to immunoprecipitate p185 Neu from LTR-Neu or SALTO cells. Specific reactivity was visualized by immunoblotting of immunoprecipitates using a Neu-specific commercial antibody (C18). Analysis of serum reactivity taken from representative 10^8^ pfu rV-*neu*T and V-wt vaccinated mice is depicted in Figure 3, Panel C. rV-*neu*T vaccination was able to induce specific anti-Neu antibodies able to immunoprecipitate the antigen from LTR-Neu and SALTO cells. Specific antibodies were detected in all rV-*neu*T vaccinated mice. Conversely, serum from V-wt vaccinated mice was not able to immunoprecipitate the antigen from LTR-Neu.

Specific antibody response to Neu was quantitatively evaluated by ELISA. As shown in Table 2, 10^8^ pfu rV-*neu*T vaccinated mice developed a significantly higher titer of anti-Neu antibodies than 10^7^ pfu rV-*neu*T and 10^6^ pfu rV-*neu*T vaccinated mice. No significant difference on anti-Neu titer antibodies was observed between the 10^7^ pfu rV-*neu*T and 10^6^ pfu rV-*neu*T dose. It is of note that anti-Neu serum titer paralleled antitumor *in vivo* activity of rV-*neu*T vaccinated mice. The administration of V-wt did not result in the induction of anti-Neu antibodies.

**Table 2:** **Immunoreactivity of rV-*neu*** **T vaccinated BALB-*neu*** **T mouse sera with Neu**

**Dose of rV- *neu *** **T**	**Number of mice with immune response/total**^**a**^	**Number of pooled sera**	**Serum titer (SD)**^b^
10^8^ pfu	8/8	5	11875^c^ (1250) p = 0.000288^d^ p = 0.0045^e^
10^7^ pfu	4/4	4	1950 (1706)
10^6^ pfu	6/6	5	3380 (3750)

^a^Immune response was determined by ELISA against LTR-Neu and NIH3T3. Optical density (O.D) of V-wt sera at 1:250 to LTR-Neu was ≤0.3. These values were considered negative. Values for rV-*neu*T serum was considered positive when specific O.D value was ≥0.5 at the 1:1000 dilution.

^b^Immune sera titers of BALB-*neu*T vaccinated mice were determined by ELISA against LTR-Neu and NIH3T3 using individual serum at different dilutions (1:1000, 1:2500, 1:12500). Sera titer represents the mean value of individual serum titer ± standard deviation (SD).

^c^Titer was estimated as the highest immune serum dilution generating a specific absorbance of 0.5 at 492 nm.

^d^10^8^ pfu vs 10^7^ pfu.

^e^10^8^ pfu vs 10^6^ pfu.

Experiments were then carried out to evaluate the isotype of the immunoglobulins (Igs) elicited by rV-*neu*T vaccination. As shown in Table 3, anti-Neu-immunoglobulins of rV-*neu*T vaccinated Balb-*neu*T mice were mainly of the IgG1 (32%), IgG2a (22%) and IgG2b (16%) isotype with a lesser amount of IgG3, IgM and IgA.

**Table 3:** **Anti-neu-immunoglobulins isotype of rV-*neu*** **T vaccinated BALB-*neu*** **T mice**

**Immunoglubulins isotype against Neu**					
**IgG1**	**IgG2a**	**IgG2b**	**IgG3**	**IgM**	**IgA**
32.52 ± 7.37^a^	22.28 ± 5.43	16.65 ± 5.54	11.5 ± 1.9	11.15 ± 1.78	5.87 ± 0.8

^a^Results are the mean of the percent (±standard deviation) of each immunoglobulins isotype relative to the total sera immunoglobulin content (at 1:1500) of 10^8^ pfu rV-*neu*T vaccinated mice.

### *In vitro* biological activity of immune sera of rV-*neu*T vaccinated mice

**Figure 4:**
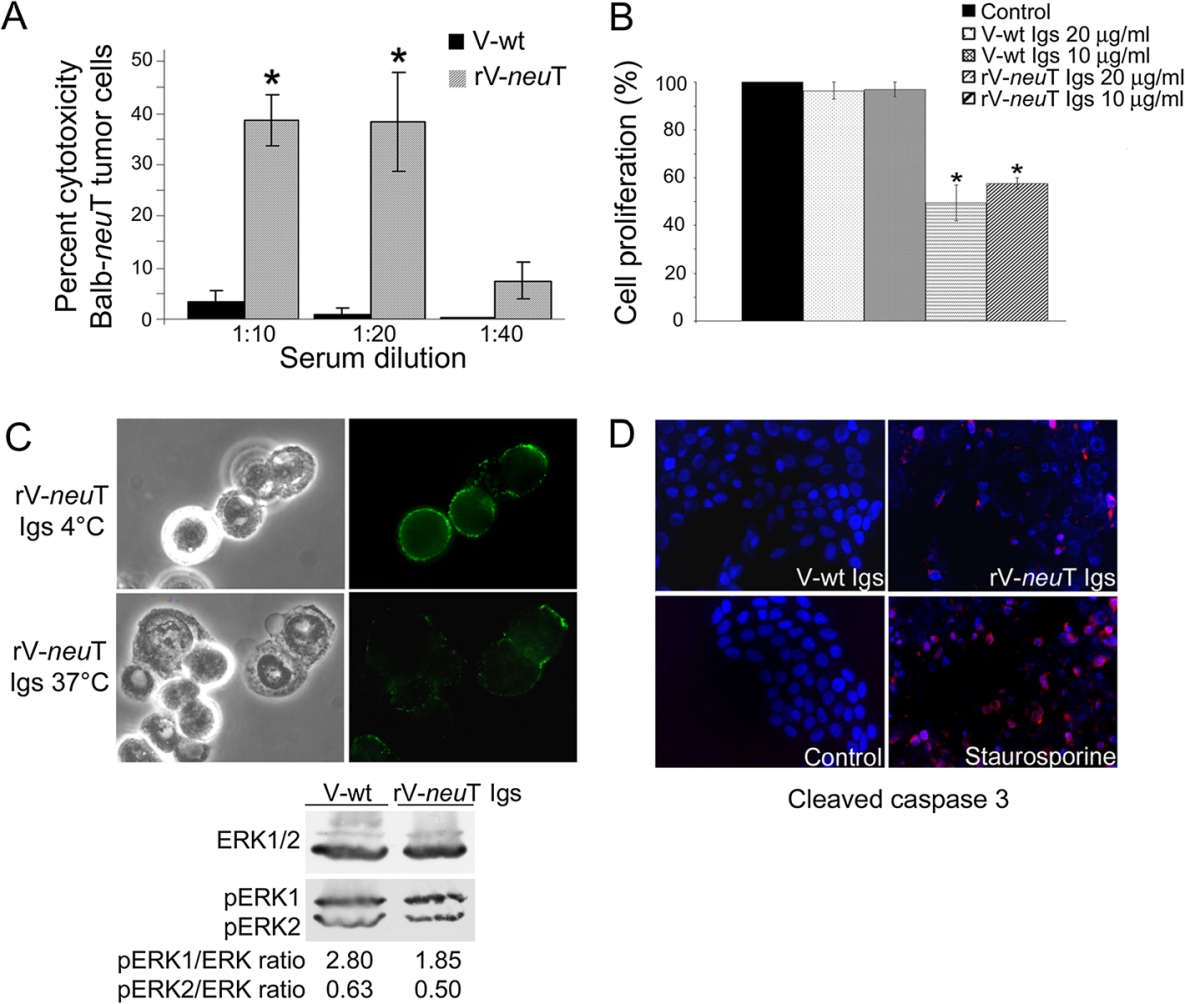
***In vitro *biological activity of immune sera or purified immunoglobulins from rV-*neu*T vaccinated mice.** Panel **A**: ADCC elicited by sera from 10^8^ pfu rV-*neu*T vaccinated mice. SALTO tumor cells were exposed for 2 hours to sera pooled from rV-*neu*T or V-wt vaccinated mice and with mononuclear effector cells derived from normal BALB/c spleens at a ratio of 50:1. Results represent average percent cytotoxicity of two independent experiments. *p ≤ 0.01. Panel **B**: Effects of anti-Neu Igs on SALTO cell proliferation. SALTO tumor cells following serum depletion were incubated in DMEM medium containing 0.2% BSA with or without rV-*neu*T or V-wt purified Igs. Relative cell numbers of triplicate experiments were determined after incubation of 72 h at 37˚C using a sulforhodamine B based proliferation assay and expressed as percent increase or decrease in comparison to vehicle control (DMEM 0.2% BSA). *p ≤ 0.01. Panel **C**: p185 Neu receptor down regulation by anti-Neu Igs. SALTO cells were stained with stained with rV-*neu*T purified Igs and then with goat anti-mouse IgG Alexa fluor-488-conjugated antibody. Original magnification x 500. Expression and phosphorylation of ERK1 and ERK2 after rV-*neu*T Igs chronically treatment of SALTO tumor cells. Serum depleted SALTO cells were treated with rV-*neu*T- and V-wt Igs and the ratio between ERK1 and ERK2 and pERK1/pERK2 expression was analyzed by western blotting. Densitometric ratios between the phosphorylated and total levels of each protein are reported. Panel **D**: Effects of anti-Neu Igs on SALTO cells apoptosis. SALTO cells were plated at 2.5×10^4^ cells/well, incubated in DMEM medium containing 0.2% BSA with or without rV-*neu*T or V-wt purified Igs (10 μg/ml) and stained with a specific anti-cleaved caspase 3 antibody. Staurosporine (1 μM) treatment was used as positive control. Nuclei were counterstained with Hoechst 33342. Original magnification x400.

ADCC, cell proliferation of BALB-*neu*T SALTO tumor cells, receptor down regulation and induction of apoptosis in SALTO cells were analyzed using pooled sera or purified Igs from10^8^ pfu rV-*neu*T or V-wt vaccinated mice in order to investigate potential mechanisms of tumor inhibition by anti-Neu Igs. As shown in Figure 4, Panel A, spleen cells produced no cytotoxicity in the presence of pooled sera from 10^8^ pfu V-wt vaccinated mice. Conversely, spleen cells in the presence of pooled sera from 10^8^ pfu rV-*neu*T vaccinated mice mediated higher ADCC (p≤0.01) at 1:10 and 1:20 dilution (38.5 and 38.25%, respectively) than sera from V-wt vaccinated mice (3 and 0.75%, respectively).

To determine whether specific anti-Neu Igs were able to interfere with *in vitro* cell growth, SALTO cells were chronically treated (72 hours) with different concentrations of purified Igs from rV-*neu*T or V-wt vaccinated mice in absence of fetal bovine serum. As shown in Figure 4, Panel B, specific inhibition of cell growth was observed after treatment with Igs from rV-*neu*T vaccinated mice both at a concentration of 20 and 10 μg/ml (50.5 and 42.5% respectively, p ≤ 0.01). Treatment with V-wt vaccinated mice Igs was not effective on cell proliferation.

Trastuzumab, a monoclonal antibodies to p185, was shown to induce down regulation of p185 receptor on cell membrane, to block its function by hampering the formation of homodimers and heterodimers and ligand binding [42]. The ability of purified Igs from vaccinated mice to induce down regulation of p185 Neu was investigated by immunofluorescence and deconvolution analysis of immunolabeled SALTO cells. SALTO cells were stained with rV-*neu*T purified Igs, then with a goat anti-mouse fluorescent antibody and incubated for 1 hour at 37°C in a CO_2_ incubator in complete medium. As shown in Figure 4, Panel C, Igs from rV-*neu*T vaccinated mice were able to induce down regulation of the p185 Neu receptor expressed on the cell surface of SALTO cells.

MAP (Mitogen Activated Protein) kinases, ERK1 and ERK2, are activated by ErbB2/Neu receptor and transduce proliferation signals. Given that chronic treatment with 10^8^ pfu rV-*neu*T Igs was able to specifically inhibit SALTO cell growth, we investigated whether phosphorylation of ERK1/ERK2 in SALTO cells was affected by rV-*neu*T Igs treatment (10 μg/ml). V-wt purified Igs were used as control. The amount of phosphorylated ERK1 and ERK2 (pERK1 and pERK2) proteins were compared to total ERK proteins. The level of total ERK1/2 did not change after 10^8^ pfu rV-*neu*T or V-wt purified Igs treatment. Conversely, phosphorylation of ERK1 was significantly inhibited by 10^8^ pfu rV-*neu*T Igs as compared to V-wt Igs treatment (ratio 1.85 vs 2.80, p = 0.011). pERK2 was only slightly inhibited (ratio 0.50 vs 0.63, p = 0.028).

To determine whether anti-Neu Igs were able to trigger apoptosis, SALTO cells were labeled with anti-activated caspase-3 polyclonal antibody after rV-*neu*T or V-wt Igs (10 μg/ml) chronic treatment. Figure 4, Panel D shows detection of cleaved caspase-3 in SALTO cells. The fraction of apoptotic cells was determined relative to cleaved caspase-3 positive cells. rV-*neu*T purified Igs induced apoptosis in 19.2% (p < 0.001) of SALTO cells. In comparison, treatment with V-wt Igs triggered irrelevant SALTO cells apoptosis (0.2%). Treatment of cells with 1 μg/ml staurosporine resulted in 60% apoptotic cells.

Overall, our results indicate that *in vitro* biological activity (ADCC, inhibition of cell proliferation, down regulation of p185 Neu receptor, inhibition of ERK1/2 phosphorylation and induction of apoptosis) of rV-*neu*T immune sera can provide the ability of rV-*neu*T vaccinated mice of interfering with tumor growth *in vivo.*

### T cell immune response induced by rV-*neu*T vaccination

Splenocytes isolated from mice vaccinated with rV-*neu*T or V-wt after the final boost, were examined for their ability to proliferate under various Neu peptides. Release of IL-2 and IFN-γ was measured in the supernatant to assess T cell immunoreactivity with specific Neu epitopes. Results are reported in Table 4. All analyzed Neu peptides, except for an unrelated gag peptide, were able to specifically activate splenocytes from rV-*neu*T immunized BALB-*neu*T mice. ConA was used as positive control.

**Table 4:** **T cell immune response of BALB-*neu*** **T mice following vaccination with rV-*neu*** **T**

**T cell *in vitro *** **stimulus**	**Neu peptide sequence**	IL-2 release*		**IFN-γ**	
		**rV-*neu*** **T**	**V-wt**	**rV-*neu*** **T**	**V-wt**
**r15.3**	TYVPANASL	208	44	519	≤18
**r41**	DMVLWKDVFRKNNQL	359	43	475	≤18
**r98**	IAPLRPEQLQVFETL	343	44	510	≤18
**r141**	LPCHPECQPQNSSET	209	43	555	≤18
**r156**	GICQPCPINCTHSCV	265	45	365	≤18
**r166**	VLLFLILVVVVGILI	239	42	585	≤18
**GAG**		35	41	3	≤18
**ConA**		1450	2800	2890	3150

*Spleen cells from vaccinated mice were stimulated *in vitro* with Neu-specific peptides. IL-2 and IFN-γ was quantitated in the supernatant (pg/ml) as a measure of T cell immunoreactivity with specific Neu epitopes. ConcanavalinA (ConA) for global T cell activation and an unrelated gag peptide served as positive and negative control, respectively.

However, the extent of IL-2 and IFN-γ release was dependent on the stimulating Neu peptide. The strongest IL-2 release was observed upon stimulation with r41 and r98 peptides which are located in the extracellular domain of rat Neu sequence. Lower IL-2 release was observed upon stimulation with r166 and r156 peptides located in the transmembrane and extracellular domains, respectively, or with r15.3 and r141 peptides. These latter are located in the extracellular domain. The strongest IFN-γ release was detected upon stimulation with r166 and r141 peptides. High levels of IFN-γ were also obtained upon r15.3 and r98 peptides. The r41 peptide, which stimulated the higher IL-2 release, induced less IFN-γ release. Within the different peptides, r156 stimulated the lowest IFN-γ release.

## Discussion

The incidence of head and neck carcinomas (HNC) is increasing worldwide and despite advances in their treatment, the survival rate of patients with this type of cancer has not substantially changed over the last two decades [43]. Salivary gland carcinomas are head and neck tumors of heterogeneous morphology that require typical surgical and adjuvant therapy [44]. Conservative surgery with nerve monitoring remains the state-of-the-art. Adjuvant radio(chemo)therapy is shown to increase local tumor control, but overall survival is not automatically enhanced [44]. Thus, the development of novel therapies can supplement the pharmaceutical armamentarium presently used for the treatment of HNC and salivary gland carcinomas. A significant tumor specific overexpression of all four ErbB receptors including EGFR, ErbB2, ErbB3, and ErbB4 has been reported in head and neck squamous cell carcinomas (HNSCC) [45,46]. ErbB2 overexpression was observed in about 20% of patients with salivary duct cancer (SDC) [47], a rare high-grade aggressive tumor subtype of salivary gland carcinoma. In agreement with both EGFR and ErbB2 overexpression, cetuximab and trastuzumab, which target EGFR and ErbB2, respectively, represent important tools for treatment of salivary gland carcinomas [48]. Indeed, it was reported that a patient with SDC positive for ErbB2 had a complete objective response after combined treatment with paclitaxel, carboplatin, and trastuzumab [49]. Similarly, it was described a case of ErbB2-positive metastatic submandibular SDC with a complete and durable clinical response after treatment with trastuzumab in combination with chemotherapy [50]. In addition, resolution of measurable and minimal residual disease in a patient with salivary duct cancer treated with trastuzumab, lapatinib, and bevacizumab, with treatment ongoing for more than 2 years was observed [51]. Thirteen patients with SDC and ErbB2 expression were treated with trastuzumab in adjuvant (n = 8) or palliative (n = 5) setting. It was reported that all patients with metastatic disease (5/5 patients) responded to treatment with trastuzumab. One patient achieved a complete response and remains with no evidence of disease 52 months after initiation of trastuzumab. The median duration of response was 18 months [52]. However, it was also reported in breast cancer patients that the objective response to trastuzumab monotherapy had a median duration of 9 months, and that the majority of responsive patients displayed resistance within 1 year [53]. Conversely, combination therapy with trastuzumab and an ErbB2/Neu T helper peptide vaccine was well tolerated and it was associated with minimal toxicity in patients with metastatic breast cancer. In addition, the combinatorial approach of the vaccine with passive immunotherapy resulted in prolonged, robust, antigen-specific immune responses in treated patients and induced epitope spreading [54]. In agreement with these evidences it is reasonable to investigate ErbB2 cancer vaccine approaches with the aim to improve the objective tumor inhibitory response in salivary gland carcinomas.

Poxvirus represents an attractive delivery vehicle of tumor antigens due to the normal post-translational modification of the inserted antigen and strong immunogenicity [2,3,5]. Engineered attenuated recombinant vaccinia virus encoding for tumor associated antigens has now been widely employed as a cancer vaccine in several clinical trials [4-9,11,13,16,18,55]. Vaccination with recombinant vaccinia virus can be achieved by systemic or local intratumoral (i.t.) injection [2,4-9,11,13,16,18].

Recently, it was demonstrated that the antitumor activity induced by i.t. vaccination with an avipox virus expressing carcinoembryonic antigen (CEA) and multiple co-stimulatory molecules was superior to that induced by systemic (subcutaneous) vaccination in CEA-transgenic mice [19]. Similarly, we recently demonstrated that local delivery of recombinant vaccinia virus encoding for *neu* (rV-*neu*T) was superior to systemic vaccination in inhibiting the neu oncogene-mediated mammary carcinogenesis [20]. Besides, i.t. injection of recombinant attenuated Salmonella enterica serovar Typhimurium vaccine has been reported to significantly inhibit Her-2/neu-expressing tumor growth. The vaccine elicited transformation of immunosuppressive myeloid-derived suppressor cells into TNF-α-secreting neutrophils and reduced the generation of Treg cells [56]. Similarly, i.t adenovirus (Ad) vaccination supported the generation of both Neu- and Ad-specific T effector cells [57]. Of note, it was reported that i.t. vaccination with vaccinia-expressing the tumor antigen HY and granulocyte macrophage colony-stimulating factor was able to overcome immunological ignorance to the tumor antigen [58]. Head and neck cancers are loco-regional diseases that appear at or close to the body surface and are easily accessible. Thus, the accessibility of salivary gland tumors allow one to envision intratumoral immunotherapy in a neoadjuvant setting.

The attempt to use intratumoral vaccination in HNC was reported by Dasgupta *et al*. [59]. In this study it was demonstrated that recombinant vaccinia virus expressing interleukin-2 invoked anti-tumor cellular immunity in an orthotopic murine model of HNC. However, no antigen was delivered by using this approach.

In this report, we investigated the efficacy of the rV-*neu*T intratumoral vaccination in hampering the growth of transplanted Neu-overexpressing salivary gland cancer cells (SALTO) in syngeneic, Neu-tolerant BALB-*neu*T mice. In addition, we determined whether the efficiency of vaccination was dependent on rV-*neu*T doses (10^8^-10^6^ pfu). Mice transgenic for the rat *neu* oncogene (BALB-*neu*T) are usually employed to evaluate the ability of ErbB2/Neu vaccines to inhibit the progression of *neu*-driven carcinogenesis [60]. Our observations indicated that the efficiency of vaccination was dose-dependent. Mice vaccinated with 10^8^ pfu rV-*neu*T had a mean survival time of 27 weeks while those receiving the 10^7^ pfu and 10^6^ pfu rV-*neu*T doses had a mean survival time of 5.25 and 9.33 weeks, respectively. rV-*neu*T vaccination at the dose of 10^8^ pfu induced regression of transplanted tumors while that at 10^6^ e and 10^7^ pfu provoked a delay in the tumor growth as compared to V-wt vaccination. The risk of developing tumors in the 10^6^ pfu and 10^7^ pfu rV-*neu*T vaccinated groups was 10.26 and 14.05 in comparison to the 10^8^ pfu rV-*neu*T vaccinated group. Overall, the mean survival time of mice vaccinated with rV-*neu*T, independently of the dose, was 14.8 weeks while of those receiving the V-wt was 2.63 weeks. It is of note that 8/9 rV-*neu*T vaccinated mice were tumor free six weeks after the first vaccination and remained in this status until the 30^th^ week. Conversely, V-wt vaccinated mice were sacrificed for exceeded tumor volume or spontaneously died at the third week after the first vaccination.

We previously established that immune response and antitumor activity were increased by repeated rV-*neu*T vaccinations. Accordingly, we performed two immunizations. One of the potential drawbacks in the use of many recombinant vaccinia immunizations in patients is that pre-existing and/or stimulated antibody and T cell response to vaccinia virus will preclude the spread of the administered vaccinia virus and thus decrease the expression of the inserted antigen. On the other hand, it should be noted that smallpox was eradicated worldwide more than 25 years ago; thus, young people are no longer vaccinated. In addition, recombinant avipox virus, which has a limited viral replication, can be employed to boost immune response after priming with recombinant vaccinia.

The extent of tumor growth interference *in vivo* was associated with high serum levels of anti-Neu antibodies, which were able to recognize p185 Neu expressed on SALTO tumor cells. 10^8^ pfu rV-*neu*T vaccinated mice developed a significantly higher titer of anti-Neu antibodies than 10^7^ and 10^6^ pfu rV-*neu*T vaccinated mice. Thus, the amount of produced anti-Neu antibodies was coincident with the efficiency of *in vivo* anti-tumor activity of rV-*neu*T vaccinated mice.

Individual mechanisms including ADCC, CDC (complement dependent cytotoxicity), induction of apoptosis, or receptor down regulation have been implicated to elucidate the inhibitory effect of anti-ErbB2/Neu antibodies on the growth of cancer cells expressing ErbB2/Neu [35,61-67]. In this study, we demonstrated that Igs from rV-*neu*T vaccinated mice inhibited *in vitro* cell proliferation, mediated ADCC and induced apoptosis of SALTO tumor cells. Indeed, immune sera from rV-*neu*T vaccinated mice were able to mediate ADCC *in vitr*o. Igs of the IgG2a isotype have been shown to mediate a more potent ADCC than other Ig isotypes in mice [68]. Anti-Neu antibodies of the IgG2a isotype are well represented in sera of rV-*neu*T vaccinated mice. Purified Igs from rV-*neu*T vaccinated mice were also able to induce inhibition of SALTO tumor cell growth.

Trastuzumab was shown to induce down regulation of p185 Neu receptor and to block receptor function [42]. We demonstrated that chronic treatment with purified rV-*neu*T Igs were able to induce down regulation of p185 Neu receptor in SALTO cells. This biological effect can make the receptor unavailable for ligands binding thus blocking its signal transduction as we observed by revealing inhibition of the MAP kinases cascade upon rV-*neu*T Igs incubation of SALTO cells. Moreover, rV-*neu*T vaccinated mice purified Igs were able to induce apoptosis of BALB-*neu*T tumor cells *in vitro*.

It has been demonstrated that cytokines and antibody production are mostly responsible for inhibition of tumor growth in BALB-*neu*T mice, while cytotoxic T lymphocytes might have a marginal role [35,69]. Here, we found that spleen T cells of rV-*neu*T vaccinated mice released IFN-γ and IL-2 upon stimulation with several Neu-specific peptides. Recognition of these epitopes *in vivo* potentially activates T cells to secrete IFN-γ thus determining ischemic necrosis at the tumor site. Such immunodominant epitopes might boost an immune response in BALB-*neu*T mice. Overall, our study suggests that rV-*neu*T i.t vaccination could be employed to induce an efficient antitumor response and reject transplanted salivary gland tumors. A Phase I study of i.t vaccine administration in men with locally recurrent or progressive prostate cancer was performed [70]. The intraprostatic administration of PSA-TRICOM [encoding transgenes for prostate-specific antigen (PSA) and 3 costimulatory molecules] poxviral vaccine was safe and feasible and could generate a significant immunologic response. Indeed, improved serum PSA kinetics and intense post-vaccination inflammatory infiltrates were seen in the majority of patients after vaccination [70]. Local vaccination with recombinant vaccinia virus might provide danger signals which can induce a specific immune response by alerting and activating specialized antigen presenting cells expressing costimulatory molecules and thus promoting T and B cell activation [71]. Active immunization targeting ErbB2 might block tumor growth more proficiently than passive immunotherapy thanks to the activation of a persistent memory immune response. It would also be useful in boosting a spontaneous occurring ErbB2 immune response. Moreover, an ErbB2 vaccine-based therapy might be helpful to a single anti-ErbB2 Mab therapy by concurrently inducing T and B cell immunity to several immunodominant epitopes.

Our findings may have a significant role for planning cancer vaccine protocols for the treatment of salivary gland tumors and other accessible tumors using i.t injection of recombinant vaccinia virus.

## Conclusions

rV-*neu*T intratumoral vaccination could be employed to induce an efficient antitumor response and reject transplanted salivary gland tumors. Our findings may have important implications in the design of cancer vaccine protocols for the treatment of salivary gland tumors and other accessible tumors using intratumoral injection of recombinant vaccinia virus.

## Abbreviations

ADCC: Antibody-dependent cell-mediated cytotoxicity; rV: Recombinant Vaccinia; ConA: Concanavalin A.

## Competing interests

The authors declare that they have no competing interests.

## Authors’ contributions

LM performed ADCC, cell proliferation, indirect immunofluorescence, immunoprecipitation and analyzed all of the results. MF carried out the statistical analysis and PCR and T cell response. MB followed the mating of mice. PS performed western blotting and analysis of apoptosis. MGG, IT, PL participated in analysis of experiments and results. FL, FC, PN, JS, AM critically revised the manuscript. RB conducted the antitumor animal experiments, carried out experimental design, supervised the project and wrote the manuscript. All authors read and approved the final manuscript.
